# Interface Architecture of a VHL-PROTAC Complex with and without Cullin‑2

**DOI:** 10.1021/jacs.6c01509

**Published:** 2026-05-06

**Authors:** Evan N. Whitford, Joshua D. Gilbert, Marius M. Kostelic, Aaron C. Ehlinger, Tiffany A. Thibaudeau, Shaun M. McLoughlin, Vicki H. Wysocki

**Affiliations:** † School of Chemistry & Biochemistry, 1372 Georgia Institute of Technology, Atlanta, Georgia 30332, United States; ‡ Native MS Guided Structural Biology Center, Georgia Institute of Technology, Atlanta, Georgia 30332, United States; § Native MS Guided Structural Biology Center, 2647 The Ohio State University, Columbus, Ohio 43210, United States; ∥ Department of Chemistry and Biochemistry, The Ohio State University, Columbus, Ohio 43210, United States; ⊥ Target Enabling Technologies, 359181 AbbVie, 1 N. Waukegan Rd, North Chicago, Illinois 60064, United States; # Technology and Therapeutic Platforms, AbbVie, 1 N. Waukegan Rd, North Chicago, Illinois 60064, United States

## Abstract

Proteolysis Targeting Chimeras (PROTACs) are bispecific molecules that link a target protein to an E3 ligase, leading to ubiquitination and subsequent degradation. Their efficacy depends on their ability to form ternary complexes for target ubiquitination, which is influenced by protein–protein interactions. Native mass spectrometry combined with surface-induced dissociation (SID) is a sensitive technique for rapidly assessing protein structures, including stoichiometry and interfacial strengths. Native mass spectrometry can also capture a variety of conformational states in the gas phase, reflecting the intrinsic flexibility of many protein assemblies. This ability to resolve structural heterogeneity and transient subpopulations provides complementary insights not as readily accessible through crystallography, cryo-EM, or other ensemble-averaging assays. By coupling native mass spectrometry with surface-induced dissociation, topological features, specifically relative interfacial strengths and subcomplex arrangements, were probed *with and without* the scaffold protein Cullin-2 added to a PROTAC-mediated ternary complex. PROTAC-mediated ternary complexes yield rich SID fragmentation into several subcomplexes. The extensive fragmentation observed for the PROTAC-assembled complex lacking Cullin-2 suggests that this Cullin-free ternary complex is more conformationally flexible, enabling multiple accessible subcomplex topologies. Although PROTACs facilitate strong, noncovalent interactions between the target protein and the E3 ligase, the addition of Cullin-2 reduced the conformational flexibility of the E3 ligase complex. This results in a pronounced reduction in fragmentation and offers critical insight into the hierarchical connectivity of the ternary complex.

## Graphical abstract

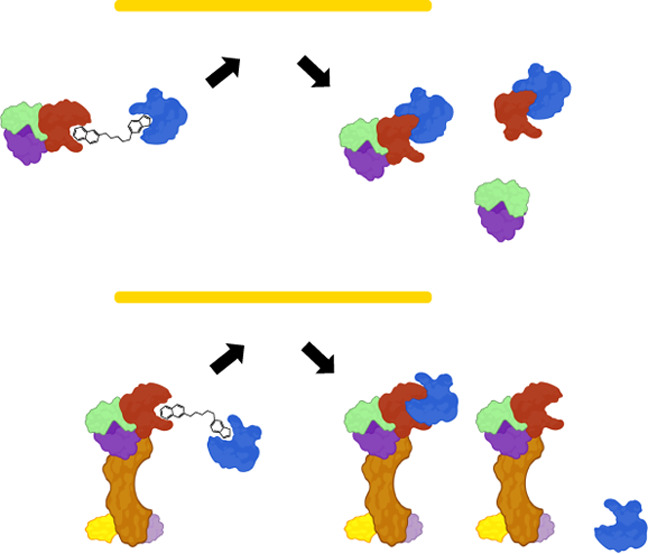

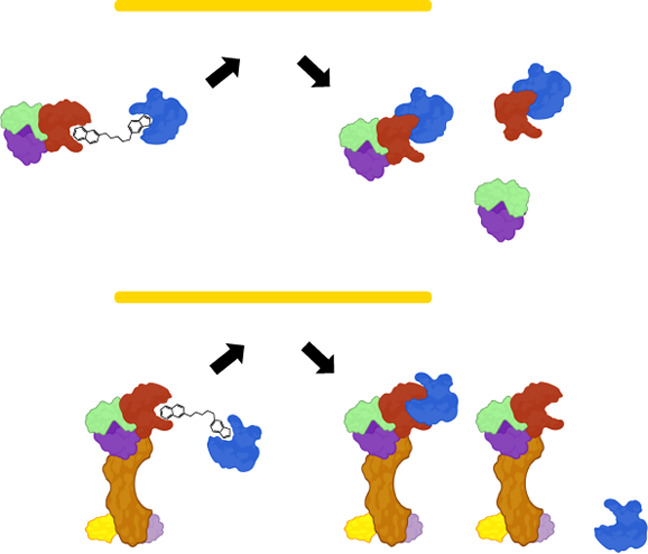

## Introduction

Targeted protein degradation is a rapidly expanding field that relies on the use of small molecules to induce prolonged proximity between proteins, causing the destruction of a target. The most common embodiment relies on recruiting E3 ubiquitin ligases to the target protein using either molecular glues or PROteolysis-TArgeting Chimeras (PROTACs). PROTACs are heterobifunctional molecules that link a target protein to an E3 ubiquitin ligase via two distinct ligands connected by a flexible linker.^1,2^ Due to its forced proximity to an E3 ligase, the target protein is tagged for degradation through polyubiquitination and subsequently broken down by the 26S proteasome, liberating the PROTAC to catalyze additional degradation events.^3^ The use of PROTACs as a therapeutic modality is attractive for target proteins previously considered undruggable, may provide selectivity between closely related protein isoforms, and may be dosed at lower concentrations owing to the catalytic recycling mechanism.^4,5^

Despite their therapeutic promise, PROTAC development is hindered by several drawbacks, notably poor physicochemical properties and unpredictable structure–activity relationships. The latter challenge arises in part from the plasticity of the ternary complex and the limited structural information available. To probe these interactions, researchers commonly employ techniques such as AlphaLISA, surface plasmon resonance, and biolayer interferometry.^6–8^ While valuable for probing binding kinetics and proximity, they often lack information on the full contingent of heteroassemblies and can suffer from incorrect fluorophore orientation, low throughput, and high resource investment.^7–11^ Techniques such as X-ray crystallography and cryo-EM offer more detailed structural insights,^12,13^ however, applying these techniques to PROTAC complexes has been challenging owing to conformational flexibility, and weak transient electrostatic interactions,^14^ which hinders the ability of the complex molecules to pack into a repeating lattice pattern^15^ or a well-defined density map.^16^ Rosetta modeling has demonstrated that PROTAC complexes exhibit a more diverse conformational landscape than those observed in crystal structures.^17^ Each of these methods contributes distinct and complementary information, and a comprehensive understanding of ternary complex behavior often requires integrating multiple orthogonal approaches.

Beveridge et al.,^18^ and Gross and coworkers^19^ were the first to apply native mass spectrometry (nMS) and native MS/MS to the structural analysis of PROTAC-mediated complexes,^20–22^ highlighting its appeal as a highly sensitive technique that requires only a few microliters of micromolar solution.^23^ Nanoelectrospray ionization^24^ based nMS is advantageous for characterizing protein complexes, as the complexes can be introduced to the instrument directly from the solution state, kinetically trapping noncovalent interactions and global structure upon transfer to the gas phase.^18,19,21,25–28^ Structural features can be elucidated by quadrupole selection followed by fragmentation^29^ and/or determination of collision cross-section values.^30^

Here, we examine the use of nMS coupled with surface-induced dissociation (SID) to characterize PROTAC-mediated complexes. A prototypical PROTAC, MZ1, binds to the target protein, bromodomain-containing protein 4 (BRD4), and the E3 ligase that ultimately binds to cullin-2 (Cul2), a scaffold protein. The E3 ligase consists of the von Hippel–Lindau (VHL, V) protein in a stable complex with elongin-B (EloB, B) and elongin-C (EloC, C), which act as adaptor proteins that bridge V to the scaffold protein Cul2, forming the VCB-Cul2 complex.^31^ On the opposite end of Cul2, NEDD8 conjugation promotes the recruitment of RING-box protein 1 (Rbx1), which in turn binds to the E2 ubiquitin-conjugating enzyme.^32^ In this architecture, V serves as the substrate recognition subunit, positioning the target protein for ubiquitination by E2-Rbx1.

In most previous work, investigators have traditionally examined ternary complexes using a variety of structural and biophysical approaches, often focusing on the minimal ***ternary*** PROTAC-mediated complex to probe intracomplex protein–protein interactions. There has been recent interest in incorporating additional components such as the scaffold protein Cul2 and its associated proteins. Sternicki et al. and Whitford et al. (this paper) both recently demonstrated that nMS can characterize Cul2-containing assemblies.^33,34^ In this work, we focus on how the presence or absence of the scaffold protein Cul2 affects complex formation, stability, and dissociation behavior to determine if any biologically relevant information (e.g., different complex dynamics) is compromised when Cul2 is not included in the system. In particular, we examine the role of SID in resolving these differences, building on prior work,^19,35^ by applying SID to probe subtle changes in complex architecture and subunit connectivity in the context of Cul2-containing and Cul2-deficient assemblies. We assess how the Cul2 scaffold modulates ternary complex behavior and whether its absence obscures mechanistically relevant features.

## Results and Discussion

### Fragmentation Differences by Gas and Surface Collisions

The PROTAC MZ1 forms a ternary complex between BRD4 and the VCB complex by tethering bromodomain 2 of BRD4 (BRD4^BD2^) to VHL through a network of noncovalent interactions.^10,36^ When MZ1 is added to a solution containing BRD4^BD2^ and the VCB complex, the BRD4^BD2^ protein and VCB protein complex assemble into a ternary complex observable by nMS.^18,19^ As shown by Gross and coworkers (and confirmed by our work) a BRD4^BD2^-VCB complex does not form without the addition of MZ1 (Figure S1). In our experiments, the BRD4^BD2^-MZ1-VCB complex was prepared under near-physiological ionic-strength conditions that reduce overall charge (160 mM ammonium acetate and 40 mM triethylammonium acetate) (Figure 1A, Figure S2).^37^ To probe its structural organization, we focused on lower charge states, which tend to favor dissociation over restructuring of protein complexes in the gas phase, and yield fragments that better reflect solution-phase topology/native architecture.^37–39^ For dissociation studies, we isolated the 10+ charge state of the ternary complex, as lower charge states typically yield more stable native-like structures and fragmentation products,^37,38^ (Figure S2) and subjected it to controlled fragmentation, enabling us to map how the complex dissociates. High-charge-state ions often favor unfolding/restructuring and non-native fragmentation pathways that obscure connectivity information.

**Figure 1:**
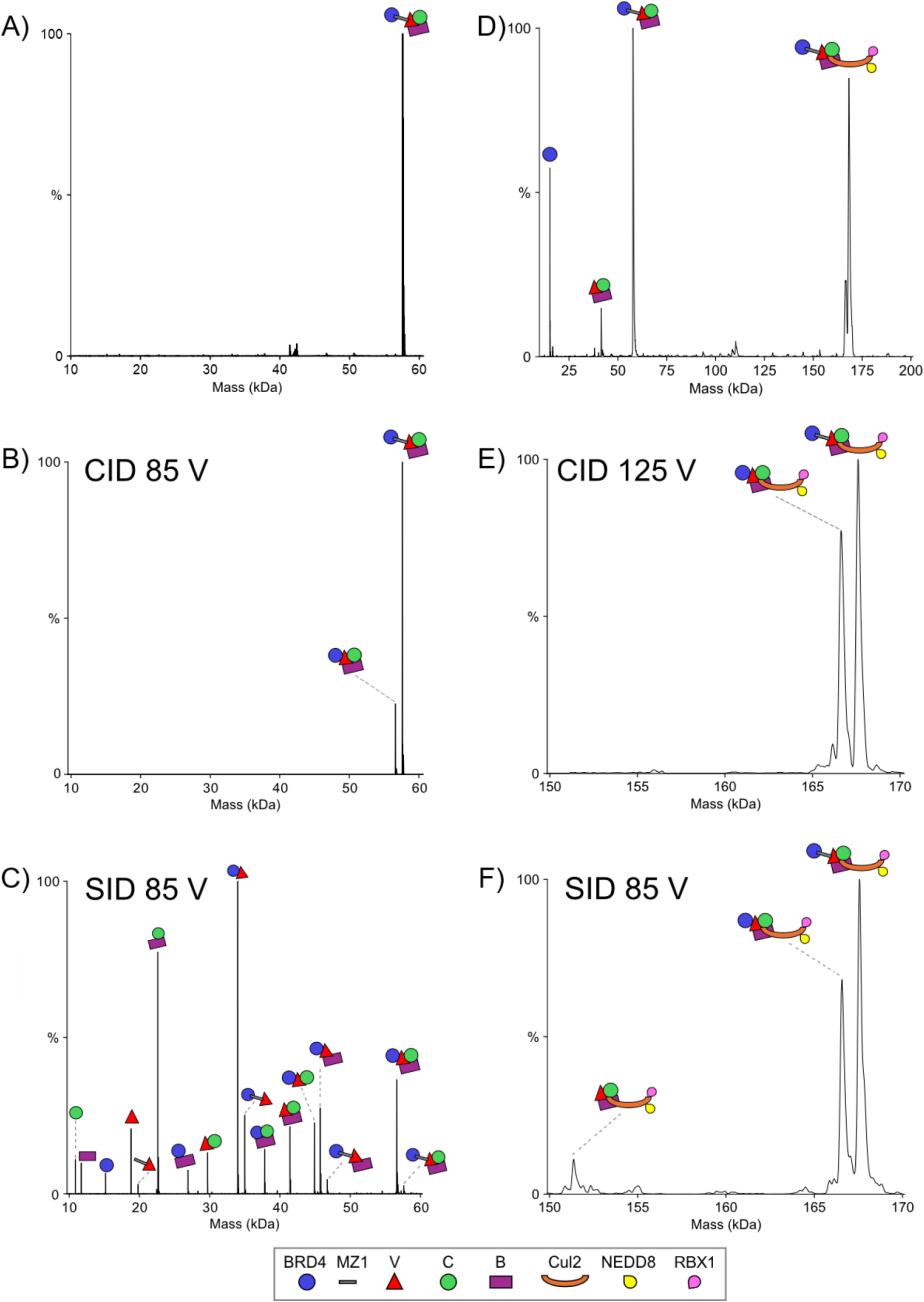
Comparison of CID and SID fragmentation in the presence and absence of Cul2. (A) Zero charge mass spectrum of 1:2:1 BRD4^BD2^-MZ1-VCB. (B) CID zero charge mass spectrum of 10+ BRD4^BD2^-MZ1-VCB (850 eV). (C) SID zero charge mass spectrum of 10+ BRD4^BD2^-MZ1-VCB (850 eV). (D) Zero charge mass spectrum of 1:2:1:1 BRD4^BD2^-MZ1-VCB-Cul2-NEDD8-RBX1. (E) CID zero charge mass spectrum of 19+ BRD4^BD2^-MZ1-VCB-Cul2-NEDD8-RBX1 (817 eV). (F) SID zero charge mass spectrum of 19+ BRD4^BD2^-MZ1-VCB-Cul2-NEDD8-RBX1 (556 eV).

Collision induced dissociation (CID) involves multiple energetic collisions with inert gas molecules, driving stepwise energy deposition that partially unfolds/restructures and fragments protein assemblies along their lowest-energy pathways. For protein complexes, this generally results in the ejection of a highly charged, elongated monomer, leaving a complementary (n–1)-mer.^23^ Under low-charge-state conditions, CID can lead to dissociation of a weakly bound subunit, yielding fragments that more closely reflect solution-phase connectivity. As precursor ions acquire higher charge, unfolding, restructuring, and the ejection of highly charged, elongated monomers occurs, leading to non-native fragmentation products.^40^ This charge-dependent behavior limits CID’s ability to unambiguously define a complex’s subunit topology.

Fragmentation of the BRD4^BD2^-MZ1-VCB complex via CID results in the ejection of the MZ1, which carries a single charge with it (Figure S3),^19^ leaving a complementary BRD4^BD2^-VCB complex (Figure 1B) as previously noted by Gross and coworkers. MZ1 ejection is observed at CID energies exceeding ∼600 eV (Figure S4). Under noncharge reducing conditions (200 mM ammonium acetate), charge states 12–15+ yield additional CID^41^ fragments in addition to MZ1 ejection (Figure S5).

With CID, ions undergo multiple collisions with gas molecules that transfer the collision energy stepwise into the protein complex, leading to restructuring and fragmentation. With SID, ions are directed to an inert surface in a single, high-energy step. This rapid energy transfer (on the order of 10^2^–10^3^ eV in the laboratory frame) often causes complexes to separate, cleaving weaker interfaces and providing valuable precursor structural information,^42^ such as substructure connectivity and relative interfacial strengths between protein subunits within a complex.^43^ In contrast to other activation methods such as ultraviolet photodissociation (UVPD), which provides complementary insights by probing local structural features within intact assemblies such as secondary structure elements and ligand binding environments,^44^ SID uniquely reports on quaternary architecture by revealing how subunits are connected and stabilized. Interfaces that are weaker cleave at lower energies, while stronger ones require more energy to dissociate. Various protein–protein interactions can be inferred through SID fragmentation.

With SID, fragmentation of BRD4^BD2^-MZ1-VCB reveals a variety of products observed at a collision potential of 85 V (850 eV for 10+), highlighting the diversity of protein–protein contacts within the assembly (Figure 1C, Figure S6). Among the dominant fragments observed are CB (∼23 kDa) and its complementary fragment, BRD4^BD2^-MZ1-V (∼35 kDa), indicating a relatively weaker interface between V and CB. This interpretation is reinforced at higher SID energies, where BRD4^BD2^-V and CB fragments become increasingly abundant (Figure 2A). Their rise in relative intensity likely reflects secondary fragmentation,^45^ which favors the retention of BRD4^BD2^-V and CB as discrete subassemblies due to their stronger internal interfaces. These patterns suggest that MZ1 not only facilitates a robust noncovalent interaction between BRD4^BD2^ and V, but also leaves CB more stably associated with each other than with V, a subtle yet informative arrangement in the complex’s architecture. This highlights a broader principle, PROTACs may not simply act as passive molecular bridges, but as architectural modulators that induce protein–protein interactions.^46^ Such induced contacts can stabilize ternary complexes, enhance cooperativity between ligase and substrate, and ultimately dictate the efficiency of targeted degradation.^47^

**Figure 2:**
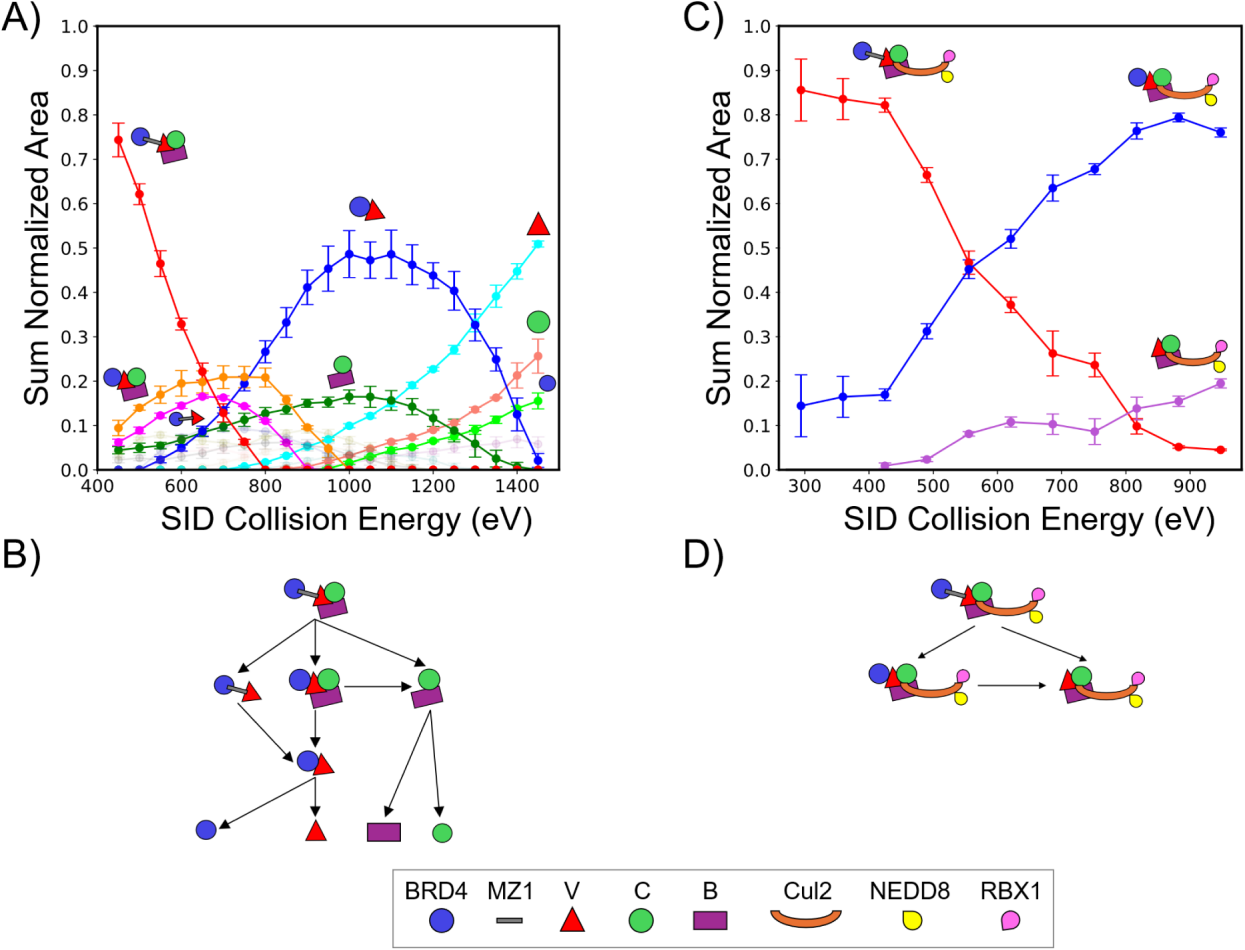
(A) ERMS plot for 10+ BRD4^BD2^-MZ1-VCB. For clarity, some fragments have a higher opacity. See Figure S11 for further data on all dissociation products. (B) Schematic branching diagram for BRD4^BD2^-MZ1-VCB depicting SID dissociation pathways. (C) ERMS plot for the higher mass, higher charge 19+ BRD4^BD2^-MZ1-VCB-Cul2-NEDD8-RBX1. The collision energies are corrected for the mass addition of Cul2, NEDD8 and RBX1. (D) Schematic branching diagram for BRD4^BD2^-MZ1-VCB-Cul2-NEDD8-RBX1 depicting dissociation pathways observed by SID.

A unique SID product occurs when MZ1-V is ejected, along with a (presumably) complementary BRD4^BD2^-CB fragment (∼38 kDa) (Figure 1C). This outcome reveals that the CB subcomplex can interface directly with BRD4^BD2^ through noncovalent contacts in the absence of Cul2, a relationship that has not been observed through crystallography. The flexibility of BRD4^BD2^ terminal regions^13^ may allow such a rearrangement to occur in solution, preserving BRD4^BD2^-CB interactions even after V is lost. There could be a solution phase rearrangement occurring, and interactions between BRD4^BD2^ and CB are being maintained. While protein ions in the gas phase can sometimes collapse into non-native conformations,^48^ we minimized this risk by using low voltage gradients^49^ to help maintain structures that closely resemble their solution-phase forms. Nevertheless, nMS alone cannot fully exclude the possibility of gas-phase artifacts, and distinguishing interactions from potential rearrangements remains an inherent limitation of the approach; comparison with the Cul2 complex (below) helps resolve this issue. Additional orthogonal structural probes, such as fast photochemical oxidation of proteins (FPOP)^50^ or related foot printing strategies,^51^ could provide complementary validation in future studies.

### Cul2 “Locks” the Multicomponent Assembly, Simplifying Dissociation Products

While these data and previous reports have focused on interfacial strengths between a minimal E3 ligase complex and its target, V acts as part of a larger multiprotein complex belonging to the family of cullin-RING ligases. These complexes not only consist of an E3 ligase and its cognate adaptor proteins, but they are further tethered to one of six cullin scaffolds, which anchor an E2 ligase through adaptor proteins, governed through neddylation. To better examine how interfacial strengths change with the larger complex, the proteins were paired with a neddylated form of Cul2 complexed with RBX1.^52^ We combined BRD4^BD2^-MZ1-VCB with neddylated Cul2-RBX1 and analyzed the resulting assemblies using nMS and nMS-SID. The native mass spectrum revealed two dominant species, the original BRD4^BD2^-MZ1-VCB ternary complex and the Cul2-bound BRD4^BD2^-MZ1-VCB-Cul2-RBX1 assembly (Figure 1D). The latter charge-reduced complexes appeared in charge states of 18+, 19+, and 20+ (Figure S7), with the 19+ charge state being the most abundant and selected for fragmentation as the 18+ lacked abundance for sufficient sampling of dissociation products. To ensure fair comparison between the Cul2-containing and Cul2-free complex, we applied mass-corrected collision energies, as previously described,^53^ to account for the difference in molecular mass. Linker ejection remained the dominant product when subjected to CID, as it was when BRD4^BD2^-MZ1-VCB underwent CID. When MZ1 is ejected from the complex by CID, it leaves a complementary BRD4^BD2^-VCB-Cul2-RBX1 subcomplex (Figure 1E, Figure S8).

When we applied SID to the Cul2-containing ternary complex, the fragmentation pattern was strikingly simpler than for the ternary complex without Cul2 (Figure 1F, Figure S9). At a collision potential of 85 V (equivalent to 555 eV for the 19+, charge state), only three species were detected, the intact precursor, a complex missing MZ1 (PROTAC ejection like CID), and a fragment missing both MZ1 and BRD4^BD2^. This sequence of events points to MZ1 as the weakest link in the Cul2-bound assembly, since its loss was the most abundant fragment. Once MZ1 was ejected, the next most vulnerable connection was the BRD4^BD2^ interface with the remaining complex. Its loss is the only other product consistently observed.

### Variation of Collision Energy Defines Relative Interface Strengths

Energy-resolved mass spectrometry (ERMS) plots track how fragment abundances change with increasing collision energy, offering a way to gauge the relative strengths of protein–protein interfaces and the overall stability of a complex.^53^ For BRD4^BD2^-MZ1-VCB, the major products observed at low SID energies are BRD4^BD2^-VCB, BRD4^BD2^-MZ1-V, and CB as noted previously (Figure 1C) and (Figure 2A). BRD4^BD2^-MZ1-V, along with its complementary fragment CB, increased with collision energy.

At higher SID energies, BRD4^BD2^-V, CB and V are more abundant. An interpretation is that while MZ1 bridges BRD4^BD2^ and VCB, its individual binding to BRD4^BD2^ and V is relatively weak. This suggests that MZ1 is no longer needed to maintain the ternary complex after it has formed. The BRD4^BD2^-V interaction, once mediated, is more stable than any other noncovalent interaction within VCB. This explains the low levels of intact VCB detected after SID. Ejection of MZ1 and loss of CB occur competitively, increasing in parallel. CB appears to dissociate both from the precursor ternary complex and from the primary MZ1-ejected fragment, whereas BRD4^BD2^-V is released through multiple fragmentation pathways (Figure 2B). The detection of individual proteins from the ternary complex (BRD4^BD2^, V, C, B) occurs only at high SID energies, indicating that dissociation requires disruption of multiple stabilizing interfaces. This extensive network of protein–protein contacts accounts for the fragment-rich spectra observed at elevated SID energies.

The ERMS plots revealed a clear difference in fragmentation patterns between the MZ1-mediated complex with and without Cul2 bound, indicating altered connectivity upon Cul2 binding. With Cul2 bound, only two fragment types appeared, MZ1 ejection followed by BRD4^BD2^ ejection (Figure 2C). At low SID energies, MZ1 ejection was the most abundant fragment in both cases. The MZ1-protein contacts are less stable than the protein–protein contacts within the complex when Cul2 is bound. This behavior suggests that MZ1’s role is primarily to initiate complex formation enabling ubiquitination. We speculate that protein–protein interfaces facilitated by MZ1 linkage remain sufficiently stable to persist without MZ1, because SID and CID produce MZ1 loss without dissociation of the protein of interest from the E3. SID fragments have previously been shown to be correlated with interface areas in protein complexes.^54^ Others have shown that PROTACs can mediate the formation of thermodynamically stable complexes even in the presence of weak target protein:PROTAC affinities (cooperativity model of PROTAC efficacy, with extensive protein–protein interactions reported between BRD4^BD2^ and VHL, mediated by MZ1).^55^ Cul2’s presence does not significantly alter the overall precursor stability, as measured by the mass-corrected collision energy required for 50% conversion of precursor to fragments (Figure S10). Without Cul2, ∼540 eV is required to fragment 50% of the precursor, compared to ∼550 eV when Cul2 is bound. This similarity reflects that the initial decay pathways dominated by MZ1 ejection are largely unaffected by Cul2 (the interfaces being broken are approximately identical). However, inspection of the alternative dissociation channels (Figure 2D) shows that once MZ1 has been removed, the residual complex is more resistant to further fragmentation when Cul2 is present. This indicates that while Cul2 does not influence the stability of the BRD4^BD2^-V interaction mediated by MZ1, it does reinforce the VCB core, consistent with Cul2-VCB contacts that persist across the energies studied. Under physiological conditions, BRD4^BD2^ and MZ1 do not naturally interact with Cul2. Structural studies show that Cul2 engages VCB through specific hydrophobic and electrostatic contacts at the VC interface, inducing a structural loop in C.^56^ These high-affinity interactions confer robustness to the ubiquitination process. In contrast, PROTAC-mediated ternary complexes, such as BRD4^BD2^-MZ1-VCB, display greater dynamic behavior prior to Cul2 binding than native protein–protein assemblies like Cul2-VCB.^57^

Taken together, the ERMS data with and without Cul2 provide complementary perspectives on the architecture and stability of the ternary complex. The ERMS analysis in the absence of Cul2 highlights the intrinsic noncovalent interactions and dynamic behavior within the ensemble, revealing that the BRD4^BD2^-V interface is the dominant stabilizing contact once MZ1 has initiated complex formation. In contrast, the Cul2-bound data underscore how recruitment of the cullin scaffold reinforces the VCB core. Thus, the two data sets converge on a coherent mechanistic picture in which PROTACs like MZ1 mediate BRD4^BD2^-VCB assembly, while Cul2 engagement locks in structure through Cul2’s high-affinity interactions with VCB. This interplay between PROTAC-induced noncovalent interactions and cullin-scaffolding illustrates how dynamic and cooperative protein–protein interfaces collectively govern the efficiency of targeted degradation.

## Conclusions

This study identifies how an MZ1 PROTAC-mediated complex organizes and stabilizes protein–protein interfaces in the presence or absence of the Cul2 scaffold protein and associated proteins. To resolve these features, we use native mass spectrometry (nMS) and surface-induced dissociation (SID) to probe structural organization and interfacial stability. For BRD4^BD2^-MZ1-VCB, CID consistently resulted in MZ1 ejection as previously reported. SID revealed MZ1 ejection and a broader range of dissociation pathways, including unique fragments not observed by CID. These fragments directly reflect the increased conformational flexibility of the ternary complex when Cul2 is absent, which enables multiple accessible dissociation pathways. Collision energy variation indicates that while MZ1 is essential for initial ternary complex formation, the BRD4^BD2^-V interface becomes the dominant stabilizing interaction postassembly. Incorporation of neddylated Cul2 into the complex altered fragmentation behavior, reducing the diversity of SID products and highlighting a sequential dissociation pathway, MZ1 ejection followed by BRD4 release, while not significantly impacting overall complex stability. The strong Cul2-VCB interface persisted across all energies studied, consistent with high-affinity native contacts that underpin E3 ligase function.

The findings in the presence and absence of Cul2 suggest that PROTAC-mediated assemblies, often examined in vitro without Cul2, exhibit conformational flexibility. Unlike single-endpoint biochemical assays or static solid-phase structures, nMS can directly capture this heterogeneity, revealing populations that other methods may average out or overlook. Such information is particularly relevant for drug design, where maximizing cooperativity and orienting ternary complexes to provide catalytic access to lysine residues is critical for efficient ubiquitin transfer. Looking forward, coupling nMS with ion mobility separations offers a promising strategy to stratify structural heterogeneity within these assemblies, providing an additional dimension of insight into how PROTACs stabilize or bias specific conformations. Data suggest that MZ1 can dissociate without disrupting the remaining ligase-substrate complex, a feature that may not be shared by all PROTACS but may be advantageous for catalytic PROTAC turnover.

Overall, the combined nMS-SID approach provides a powerful means to dissect the architecture, stability hierarchy, and dynamic behavior of PROTAC-mediated complexes, offering mechanistic insights that can inform the rational design of next-generation PROTACs with optimized stability and functional profiles. These results and this approach also lay the groundwork for future studies exploring how variations in linker chemistry, binding affinity, and BRD4-targeting ligands influence complex formation, stability, and dissociation pathways. The ease of including a cullin protein in nMS studies, and the scaffolding provided by the presence of cullin, are advantages that support inclusion of nMS in the PROTAC assessment toolbox.

## Supplementary Material



## Notes

Additional data and figures: voltages used for SID experiments (Table S1); native mass spectrum of BRD4^BD2^-VCB (Figure S1); native mass spectrum of BRD4^BD2^-MZ1-VCB (Figure S2); native mass spectrum of BRD4^BD2^-MZ1-VCB subjected to CID (Figure S3); CID-ERMS plot for 10+ BRD4^BD2^-MZ1-VCB (Figure S4); CID-ERMS plots for 10–15+ BRD4^BD2^-MZ1-VCB (Figure S5); native mass spectrum of BRD4^BD2^-MZ1-VCB subjected to SID (Figure S6); native mass spectrum of BRD4^BD2^-MZ1-VCB-Cul2-NEDD8-RBX1 (Figure S7); native mass spectrum of BRD4^BD2^-MZ1-VCB-Cul2-NEDD8-RBX1 subjected to CID (Figure S8); native mass spectrum of BRD4^BD2^-MZ1-VCB-Cul2-NEDD8-RBX1 subjected to SID (Figure S9); precursor decay plot for 10+ BRD4^BD2^-MZ1-VCB and 19+ BRD4^BD2^-MZ1-VCB-Cul2-NEDD8-RBX1 (Figure S10); SID-ERMS plot for 10+ BRD4^BD2^-MZ1-VCB with no faded fragments and SID-ERMS plot for 19+ BRD4^BD2^-MZ1-VCB-Cul2-NEDD8-RBX1 (Figure S11); detailed experimental methods covering protein expression and purification of BRD4^BD2^, VCB and Cul2 complexed with NEDD8 and RBX1; sample preparation for nMS experiments; nMS experimental parameters (PDF)

## References

[1] Lu D., Yu X., Lin H., Cheng R., Monroy E. Y., Qi X., Wang M. C., Wang J. (2022). Applications of Covalent Chemistry
in Targeted Protein Degradation. Chem. Soc.
Rev..

[2] Sakamoto K. M., Kim K. B., Kumagai A., Mercurio F., Crews C. M., Deshaies R. J. (2001). Protacs: Chimeric Molecules That Target Proteins to
the Skp1–Cullin–F Box Complex for Ubiquitination and
Degradation. Proc. Natl. Acad. Sci. U. S. A..

[3] Liu Z., Hu M., Yang Y., Du C., Zhou H., Liu C., Chen Y., Fan L., Ma H., Gong Y., Xie Y. (2022). An Overview of PROTACs: A Promising Drug Discovery Paradigm. Mol. Biomed..

[4] Wells J. A., McClendon C. L. (2007). Reaching for High-Hanging Fruit in
Drug Discovery at
Protein–Protein Interfaces. Nature.

[5] Toure M., Crews C. M. (2016). Small-Molecule PROTACS:
New Approaches to Protein Degradation. Angew.
Chem. Int. Ed..

[6] Simard J. R., Lee L., Vieux E., Improgo R., Tieu T., Phillips A. J., Fisher S. L., Pollock R. M., Park E. (2021). High-Throughput Quantitative
Assay Technologies for Accelerating the Discovery and Optimization
of Targeted Protein Degradation Therapeutics. SLAS Discovery.

[7] Roy M. J., Winkler S., Hughes S. J., Whitworth C., Galant M., Farnaby W., Rumpel K., Ciulli A. (2019). SPR-Measured
Dissociation Kinetics of PROTAC Ternary Complexes Influence Target
Degradation Rate. ACS Chem. Biol..

[8] Wang H., Zhou R., Xu F., Yang K., Zheng L., Zhao P., Shi G., Dai L., Xu C., Yu L., Li Z., Wang J., Wang J. (2023). Beyond Canonical PROTAC:
Biological Targeted Protein Degradation (bioTPD). Biomater. Res..

[9] Zorba A., Nguyen C., Xu Y., Starr J., Borzilleri K., Smith J., Zhu H., Farley K. A., Ding W., Schiemer J. (2018). Delineating the Role
of Cooperativity in the
Design of Potent PROTACs for BTK. Proc. Natl.
Acad. Sci. U. S. A..

[10] Gadd M. S., Testa A., Lucas X., Chan K.-H., Chen W., Lamont D. J., Zengerle M., Ciulli A. (2017). Structural Basis of
PROTAC Cooperative Recognition for Selective Protein Degradation. Nat. Chem. Biol..

[11] Casement, R. ; Bond, A. ; Craigon, C. ; Ciulli, A. Mechanistic and Structural Features of PROTAC Ternary Complexes. In Targeted Protein Degradation; Springer, 2021, pp. 79–113. DOI: 10.1007/978-1-0716-1665-9_5. 34432240

[12] Podobnik M., Kraševec N., Zavec A. B., Naneh O., Flašker A., Caserman S., Hodnik V., Anderluh G. (2016). How to Study Protein-Protein
Interactions. Acta Chim. Slov..

[13] Crowe C., Nakasone M. A., Chandler S., Craigon C., Sathe G., Tatham M. H., Makukhin N., Hay R. T., Ciulli A. (2024). Mechanism
of Degrader-Targeted Protein Ubiquitinability. Sci. Adv..

[14] Haubrich K., Spiteri V. A., Farnaby W., Sobott F., Ciulli A. (2023). Breaking Free
from the Crystal Lattice: Structural Biology in Solution to Study
Protein Degraders. Curr. Opin. Struct. Biol..

[15] Wagner U., Kratky C. (2015). Structure Elucidation
of Natural Compounds by X-Ray
Crystallography. Prog. Chem. Org. Nat. Prod..

[16] Bai X., Gonen T., Gronenborn A. M., Perrakis A., Thorn A., Yang J. (2024). Challenges and Opportunities
in Macromolecular Structure Determination. Nat.
Rev. Mol. Cell Biol..

[17] Bai N., Miller S. A., Andrianov G. V., Yates M., Kirubakaran P., Karanicolas J. (2021). Rationalizing PROTAC-Mediated Ternary Complex Formation
Using Rosetta. J. Chem. Inf. Model..

[18] Beveridge R., Kessler D., Rumpel K., Ettmayer P., Meinhart A., Clausen T. (2020). Native Mass Spectrometry Can Effectively Predict PROTAC
Efficacy. ACS Cent. Sci..

[19] Song J. H., Wagner N. D., Yan J., Li J., Huang R. Y.-C., Balog A. J., Newitt J. A., Chen G., Gross M. L. (2021). Native
Mass Spectrometry and Gas-Phase Fragmentation Provide Rapid and in-Depth
Topological Characterization of a PROTAC Ternary Complex. Cell Chem. Biol..

[20] Hao Y., Zhang B., Chen R. (2025). Application
of Mass Spectrometry
for the Advancement of PROTACs. J. Pharm. Biomed.
Anal..

[21] Huang X., Kamadurai H., Siuti P., Ahmed E., Bennett J. L., Donald W. A. (2023). Oligomeric
Remodeling by Molecular Glues Revealed Using
Native Mass Spectrometry and Mass Photometry. J. Am. Chem. Soc..

[22] Maciel E. V. S., Eisert J., Müller J., Habeck T., Lermyte F. (2025). Mass Spectrometry
Analysis of Chemically and Collisionally Dissociated Molecular Glue-
and PROTAC-Mediated Protein Complexes Informs on Disassembly Pathways. J. Am. Soc. Mass Spectrom..

[23] Snyder D. T., Harvey S. R., Wysocki V. H. (2022). Surface-Induced
Dissociation Mass
Spectrometry as a Structural Biology Tool. Chem.
Rev..

[24] Jordan J. S., Xia Z., Williams E. R. (2022). Tips on Making Tiny
Tips: Secrets to Submicron Nanoelectrospray
Emitters. J. Am. Soc. Mass Spectrom..

[25] Sternicki L. M., Nonomiya J., Liu M., Mulvihill M. M., Quinn R. J. (2021). Native Mass Spectrometry for the Study of PROTAC GNE-987-Containing
Ternary Complexes. ChemMedchem.

[26] Ahmed I. M. M., Beveridge R. (2023). Native Mass Spectrometry Interrogation of Complexes
Formed during Targeted Protein Degradation. Rapid Commun. Mass Spectrom..

[27] Jackson C., Beveridge R. (2024). Native Mass
Spectrometry of Complexes Formed by Molecular
Glues Reveals Stoichiometric Rearrangement of E3 Ligases. Analyst.

[28] Leney A. C., Heck A. J. R. (2017). Native Mass Spectrometry:
What Is in the Name?. J. Am. Soc. Mass Spectrom..

[29] Vallejo D. D., Rojas Ramírez C., Parson K. F., Han Y., Gadkari V. V., Ruotolo B. T. (2022). Mass Spectrometry Methods for Measuring Protein Stability. Chem. Rev..

[30] Turzo S. B. A., Seffernick J. T., Rolland A. D., Donor M. T., Heinze S., Prell J. S., Wysocki V. H., Lindert S. (2022). Protein Shape
Sampled by Ion Mobility Mass Spectrometry Consistently Improves Protein
Structure Prediction. Nat. Commun..

[31] Cardote T. A. F., Gadd M. S., Ciulli A. (2017). Crystal Structure
of the Cul2-Rbx1-EloBC-VHL
Ubiquitin Ligase Complex. Structure.

[32] Duda D. M., Borg L. A., Scott D. C., Hunt H. W., Hammel M., Schulman B. A. (2008). Structural Insights
into NEDD8 Activation of Cullin-RING
Ligases: Conformational Control of Conjugation. Cell.

[33] Sternicki, L. M. ; Crowe, C. ; Ciulli, A. ; Poulsen, S.-A. Native Mass Spectrometry Analysis of a Cullin RING Ubiquitin E3 Ligase Complex in the Context of Targeted Protein Degradation. bioRxiv 2025. 10.1021/acs.analchem.6c01970 PMC13373920 42378227

[34] Whitford, E. ; Gilbert, J. ; Kostelic, M. ; Ehlinger, A. ; Thibaudeau, T. ; McLoughlin, S. ; Wysocki, V. Interface Architecture of a VHL-PROTAC Complex With and Without Cullin-2. ChemRxiv 2025. 10.1021/jacs.6c01509 PMC13195672 42089481

[35] Gadallah M. I., Nonhof K. L., Nayak D., Zhang P., Dioli O., Zheng G., Olsen S. K., Zhou D., Brodbelt J. S. (2026). Unveiling
BCL-xL-Specific PROTAC Efficiency and Dissociation Pathways Using
Native Mass Spectrometry. Chem. Sci..

[36] Wijaya, A. J. ; Farnaby, W. ; Ciulli, A. Chapter Eleven - Crystallization of VHL-Based PROTAC-Induced Ternary Complexes. In Methods in Enzymology; Elsevier, 2023,Vol. 681,pp. 241–263. DOI: 10.1016/bs.mie.2022.10.005. 36764760

[37] Zhou M., Dagan S., Wysocki V. H. (2013). Impact
of Charge State on Gas-Phase
Behaviors of Noncovalent Protein Complexes in Collision Induced Dissociation
and Surface Induced Dissociation. Analyst.

[38] Hall Z., Politis A., Bush M. F., Smith L. J., Robinson C. V. (2012). Charge-State
Dependent Compaction and Dissociation of Protein Complexes: Insights
from Ion Mobility and Molecular Dynamics. J.
Am. Chem. Soc..

[39] Shaw J. B., Harvey S. R., Du C., Xu Z., Edgington R. M., Olmedillas E., Saphire E. O., Wysocki V. H. (2024). Protein
Complex
Heterogeneity and Topology Revealed by Electron Capture Charge Reduction
and Surface Induced Dissociation. ACS Cent.
Sci..

[40] Jia M., Song Y., Du C., Wysocki V. H. (2023). Oxidized and Reduced
Dimeric Protein Complexes Illustrate Contrasting CID and SID Charge
Partitioning. J. Am. Soc. Mass Spectrom..

[41] Hall Z., Hernández H., Marsh J. A., Teichmann S. A., Robinson C. V. (2013). The Role of Salt Bridges, Charge Density, and Subunit
Flexibility in Determining Disassembly Routes of Protein Complexes. Structure.

[42] Zhou M., Wysocki V. H. (2014). Surface Induced
Dissociation: Dissecting Noncovalent
Protein Complexes in the Gas Phase. Acc. Chem.
Res..

[43] Quintyn R. S., Yan J., Wysocki V. H. (2015). Surface-Induced Dissociation of Homotetramers with
D2 Symmetry Yields Their Assembly Pathways and Characterizes the Effect
of Ligand Binding. Chem. Biol..

[44] Brodbelt J. S., Morrison L. J., Santos I. (2020). Ultraviolet
Photodissociation Mass
Spectrometry for Analysis of Biological Molecules. Chem. Rev..

[45] Stiving A. Q., Van Aernum Z. L., Busch F., Harvey S. R., Sarni S. H., Wysocki V. H. (2019). Surface-Induced
Dissociation: An Effective Method for
Characterization of Protein Quaternary Structure. Anal. Chem..

[46] Wurz R. P., Rui H., Dellamaggiore K., Ghimire-Rijal S., Choi K., Smither K., Amegadzie A., Chen N., Li X., Banerjee A. (2023). Affinity
and Cooperativity Modulate Ternary Complex Formation to Drive Targeted
Protein Degradation. Nat. Commun..

[47] Arya, R. ; Shanavas, R. S. ; Kundu, D. ; Soudagar, M. K. ; Sanga, V. K. ; Jha, S. ; Karole, A. M. Surface Plasmon Resonance-Based Characterization of PROTAC-Induced Ternary Complexes Involving CDK2 and CRBN-DDB1. Int. J. Pharm. Sci. 2025, 3(7).

[48] Eldrid C., Cragnolini T., Ben-Younis A., Zou J., Raleigh D. P., Thalassinos K. (2022). Linking Gas-Phase
and Solution-Phase Protein Unfolding
via Mobile Proton Simulations. Anal. Chem..

[49] Arslanian A. J., Wysocki V. H. (2025). Roughhousing with Ions: Surface-Induced Dissociation
and Electron Capture Dissociation as Diagnostics of Q-Cyclic IMS-TOF
Instrument Tuning Gentleness. J. Am. Soc. Mass
Spectrom..

[50] Johnson D. T., Di Stefano L. H., Jones L. M. (2019). Fast Photochemical Oxidation of Proteins
(FPOP): A Powerful Mass Spectrometry–Based Structural Proteomics
Tool. J. Biol. Chem..

[51] Tremblay C. Y., Kirsch Z. J., Vachet R. W. (2022). Epitope
Mapping with Diethylpyrocarbonate
Covalent Labeling-Mass Spectrometry. Anal. Chem..

[52] Liu X., Zurlo G., Zhang Q. (2020). The Roles of Cullin-2 E3 Ubiquitin
Ligase Complex in Cancer. Adv. Exp. Med. Biol..

[53] Sarni S. H., Roca J., Du C., Jia M., Li H., Damjanovic A., Małecka E. M., Wysocki V. H., Woodson S. A. (2022). Intrinsically
Disordered Interaction Network in an RNA Chaperone Revealed by Native
Mass Spectrometry. Proc. Natl. Acad. Sci. U.
S. A..

[54] Harvey S. R., Seffernick J. T., Quintyn R. S., Song Y., Ju Y., Yan J., Sahasrabuddhe A. N., Norris A., Zhou M., Behrman E. J., Lindert S., Wysocki V. H. (2019). Relative Interfacial
Cleavage Energetics of Protein Complexes Revealed by Surface Collisions. Proc. Natl. Acad. Sci. U. S. A..

[55] Li K., Crews C. M. (2022). PROTACs: Past, Present
and Future. Chem. Soc. Rev..

[56] Cai W., Yang H. (2016). The Structure and Regulation
of Cullin 2 Based E3 Ubiquitin Ligases
and Their Biological Functions. Cell Div..

[57] Wu K. Y., Hung T. I., Chang C.-E. A. (2025). PROTAC-induced
protein structural
dynamics in targeted protein degradation. bioRxiv.

