# Supplementary material for: Interface Architecture of a VHL-PROTAC Complex with and without Cullin‑2
Source: J Am Chem Soc. 2026 May 6;148(19):19730–7. doi: 10.1021/jacs.6c01509 (PMC13195672; doi:10.1021/jacs.6c01509)
Supplement: Supplementary file 1 [file ja6c01509_si_001.pdf]

Supporting Information

Interface Architecture of a VHL-PROTAC Complex With and Without Cullin-2

Evan N. Whitford[1,2], Joshua D. Gilbert[3], Marius M. Kostelic[3,4], Aaron C. Ehlinger[5], Tiffany A. Thibaudeau[5], Shaun M. McLoughlin[6], Vicki H. Wysocki[1,2]

[1]School of Chemistry & Biochemistry, Georgia Institute of Technology, Atlanta, Georgia, 30332, United States.

[2]Native MS Guided Structural Biology Center, Georgia Institute of Technology, Atlanta, Georgia, 30332, United States

[3]Native MS Guided Structural Biology Center, The Ohio State University, Columbus, Ohio, 43210, United States.

[4]Department of Chemistry and Biochemistry, The Ohio State University, Columbus, Ohio, 43210, United States.

[5]Target Enabling Technologies, AbbVie, 1 N. Waukegan Rd. North Chicago, IL, 60064, United States.

[6]Technology and Therapeutic Platforms, AbbVie, 1 N. Waukegan Rd. North Chicago, IL, 60064, United States.

Lens	Transmission	SID 45 (V)	SID 45 + x (V)
Bent flatapole	5	5	5
Entrance 1-3	3	-5	-5
Entrance 2	-26	2	2
Front top	0	-23.5	-23.5+ 0.05x
Front bottom	0	-9.5	-9.5 + 0.05x
Surface	0	-40	-40 -x
Middle bottom	0	-110	-110 -x
Back top	0	-154	-154 -x
Back bottom	0	-90	-90 -x
Exit 1-3	-2	-50	-50 -x
Exit 2	-26	-30	-30 -x
C-trap entrance inject	1.8	-29	-29 -x
C-trap exit inject	-16	-20	-20 -x
C-trap offset	0	-35	-35 -x

Table S1. Voltages used for SID experiments on a modified Q Exactive Ultra High Mass Range (UHMR) Orbitrap Mass Spectrometer. SID voltage is defined by the bias between the bent flatapole and surface in the SID device.

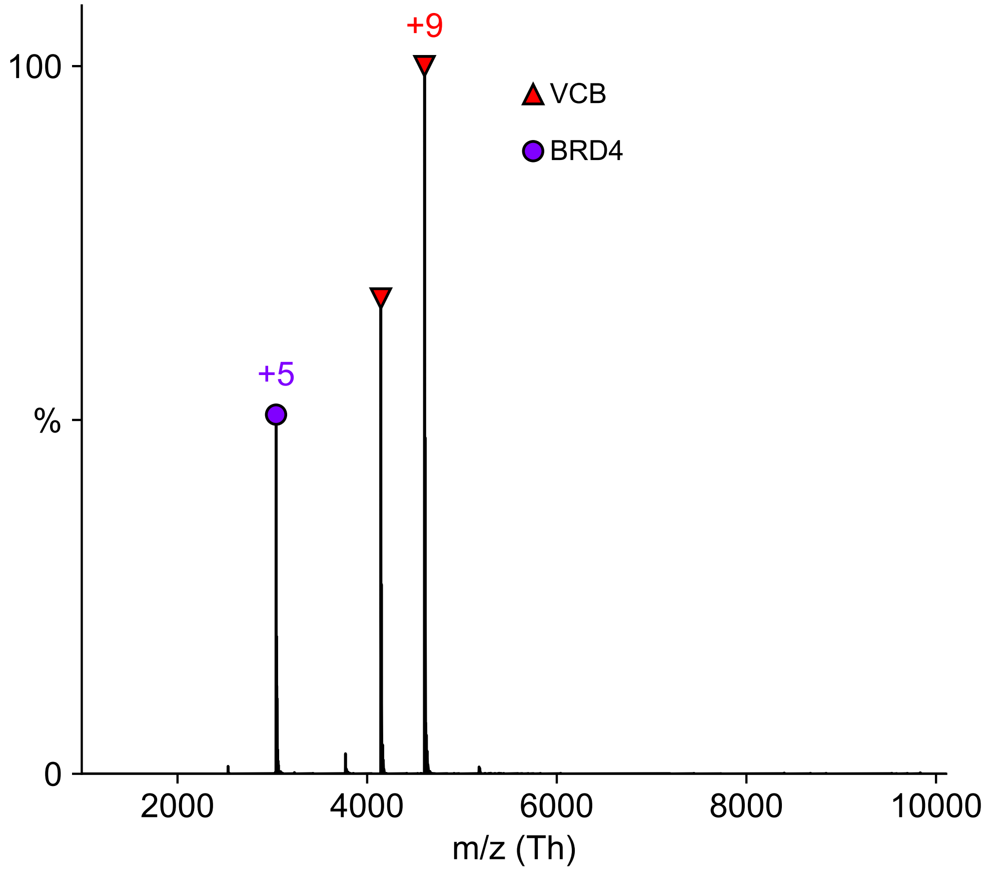

Figure S1. Native mass spectrum of 1:1 BRD4^{BD2}-VCB.

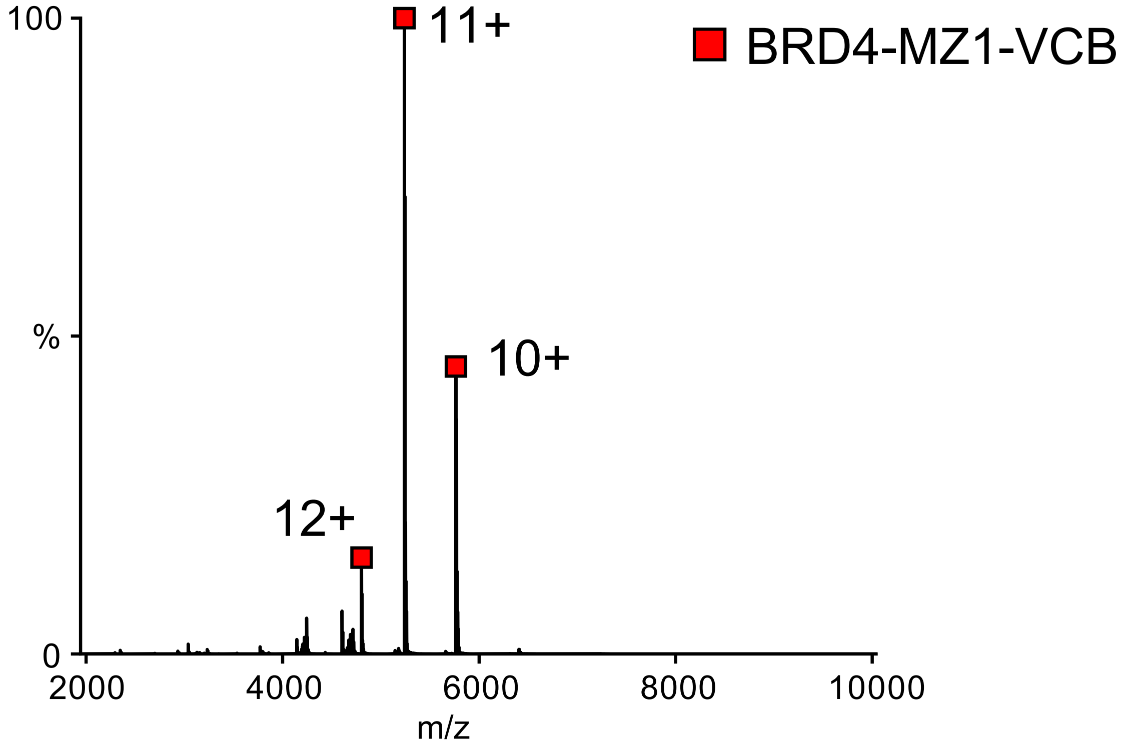

Figure S2. Native mass spectrum of 1:2:1 BRD4^{BD2}-MZ1-VCB.

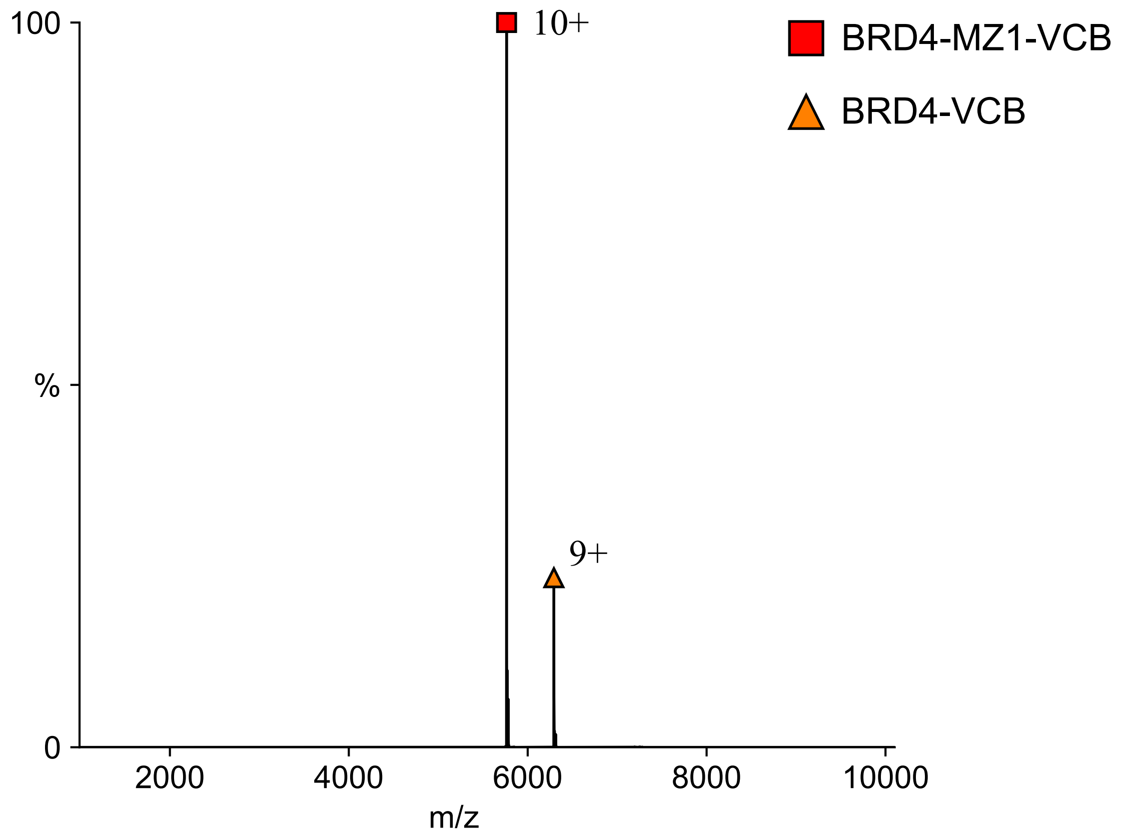

Figure S3. CID spectrum of 10+ BRD4^{BD2}-MZ1-VCB.

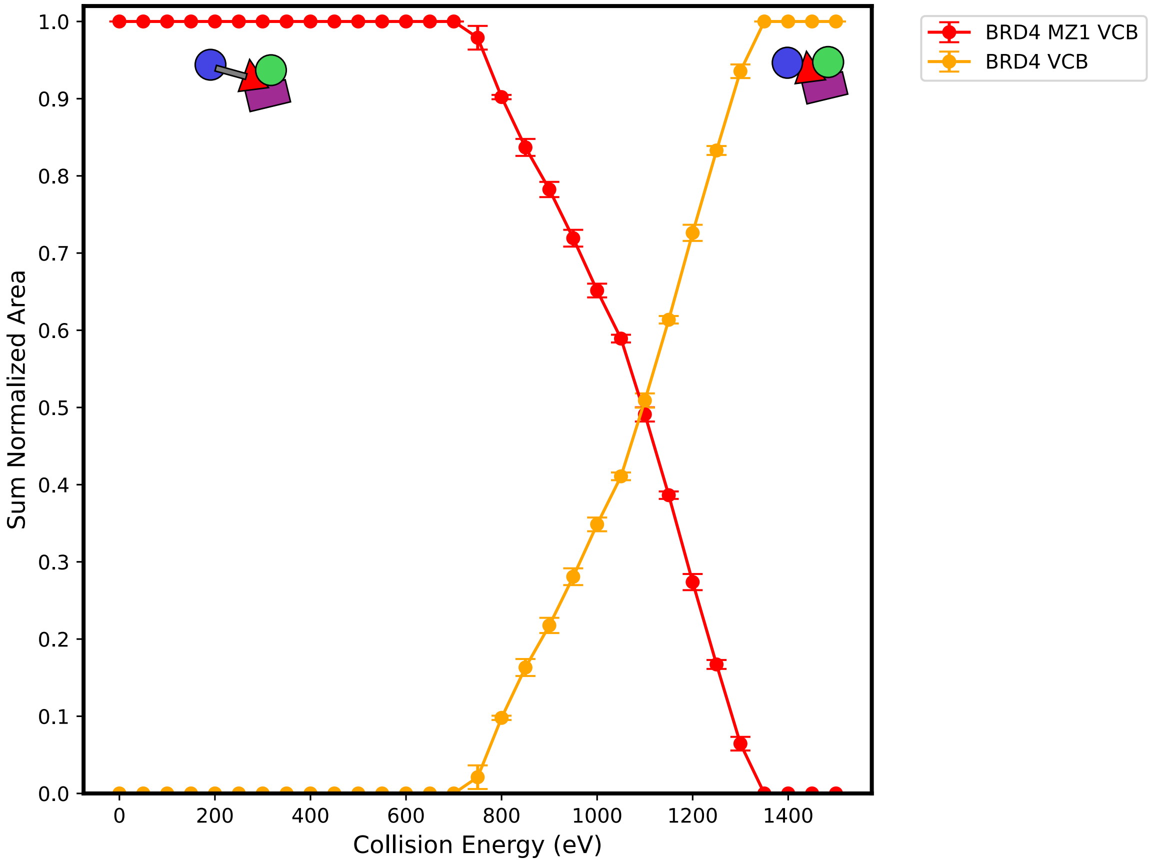

Figure S4. CID-ERMS plots for the 10+ charge state of BRD4-MZ1-VCB. The red line depicts BRD4-MZ1-VCB precursor and orange line depicts BRD4-VCB fragment which is observed after ejection of MZ1.

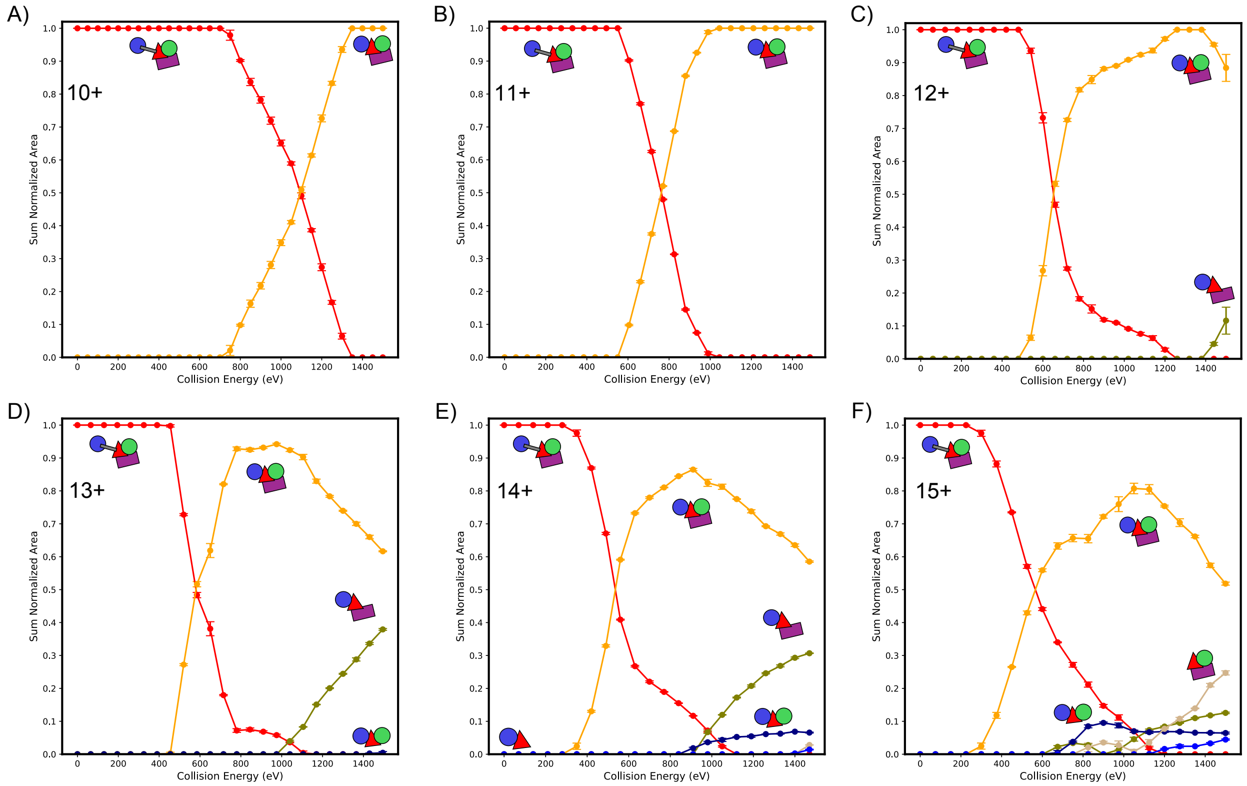

Figure S5. CID-ERMS plots of different charge states of BRD4^{BD2}-MZ1-VCB (A) 10+, (B) 11+, (C) 12+, (D) 13+, (E) 14+, (F) 15+. Red line corresponds to BRD4^{BD2}-MZ1-VCB. Orange line corresponds to BRD4^{BD2}-VCB. Olive line corresponds to BRD4^{BD2}-VB. Navy blue line corresponds to BRD4^{BD2}-VC. Royal blue line corresponds to BRD4^{BD2}-V. Tan line corresponds to VCB.

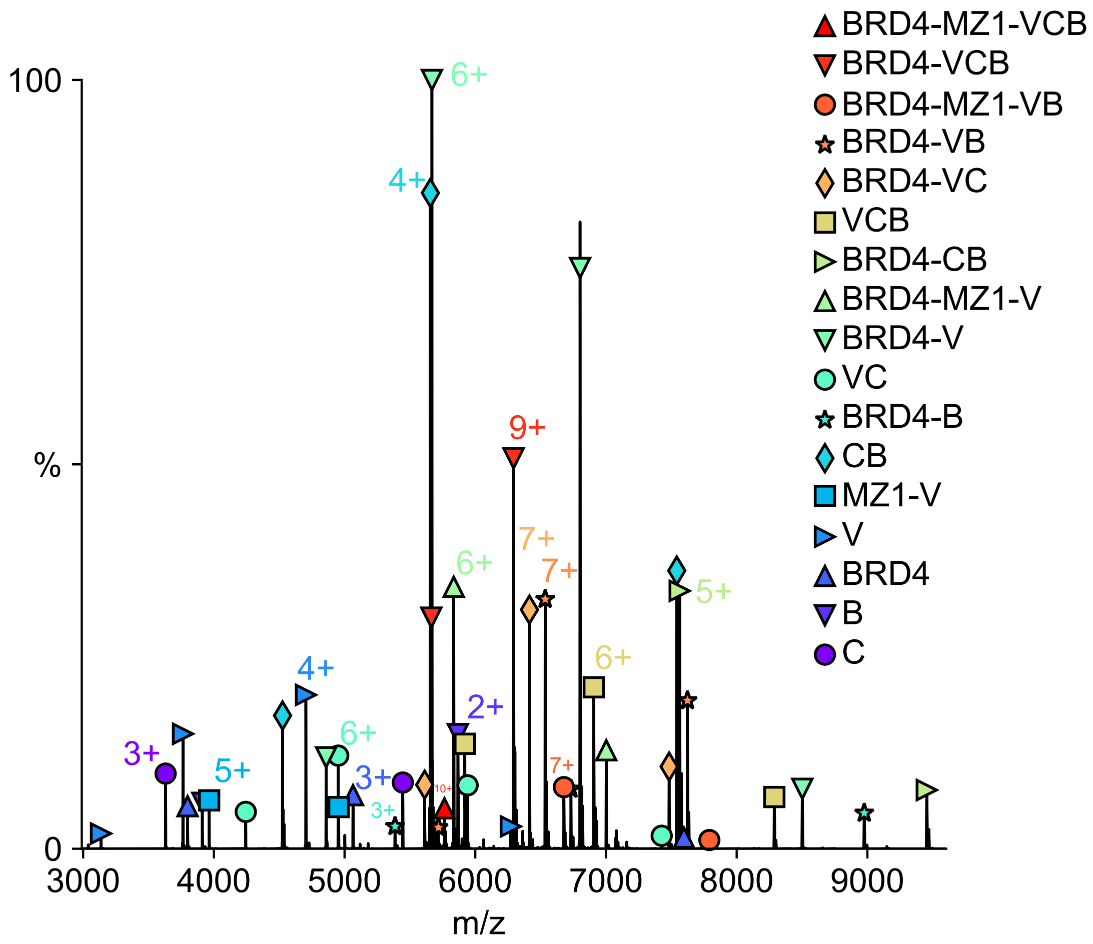

Figure S6. SID spectrum of 10+ BRD4^{BD2}-MZ1-VCB. A charge state has been assigned for each species present.

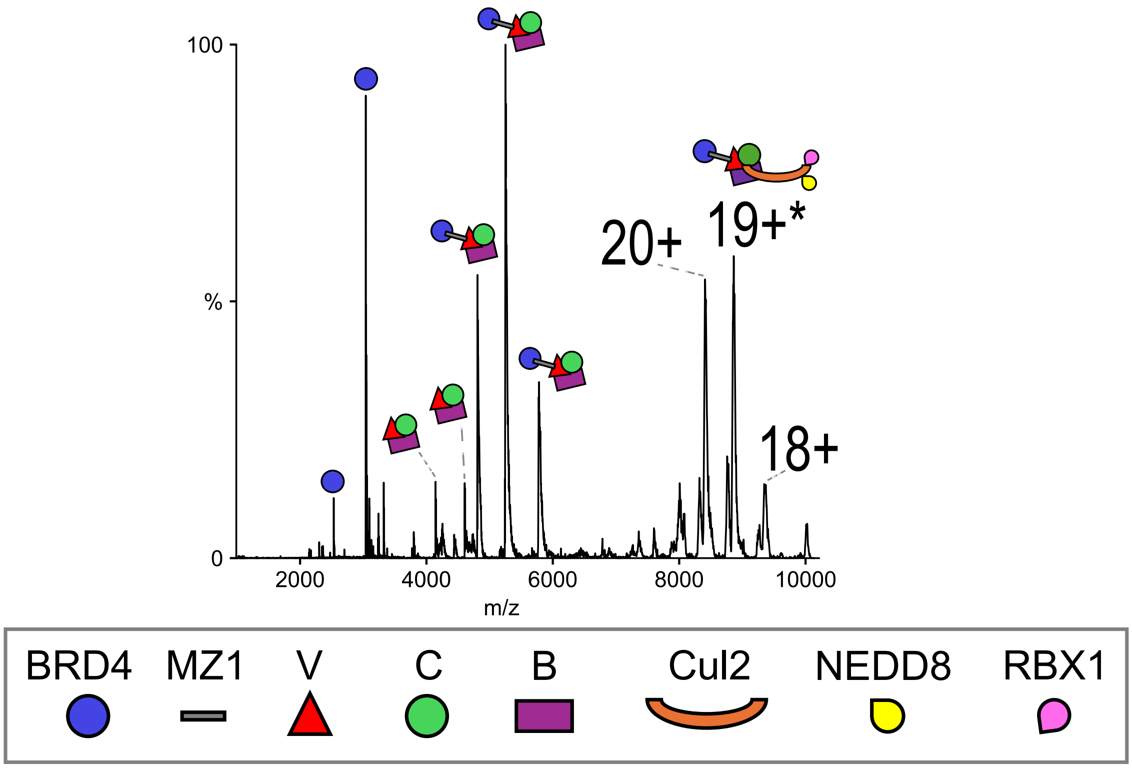

Figure S7. Native mass spectrum of 1:2:1:1 BRD4^{BD2}-MZ1-VCB-Cul2-NEDD8-RBX1. An asterisk denotes the isolated charge state for fragmentation experiments.

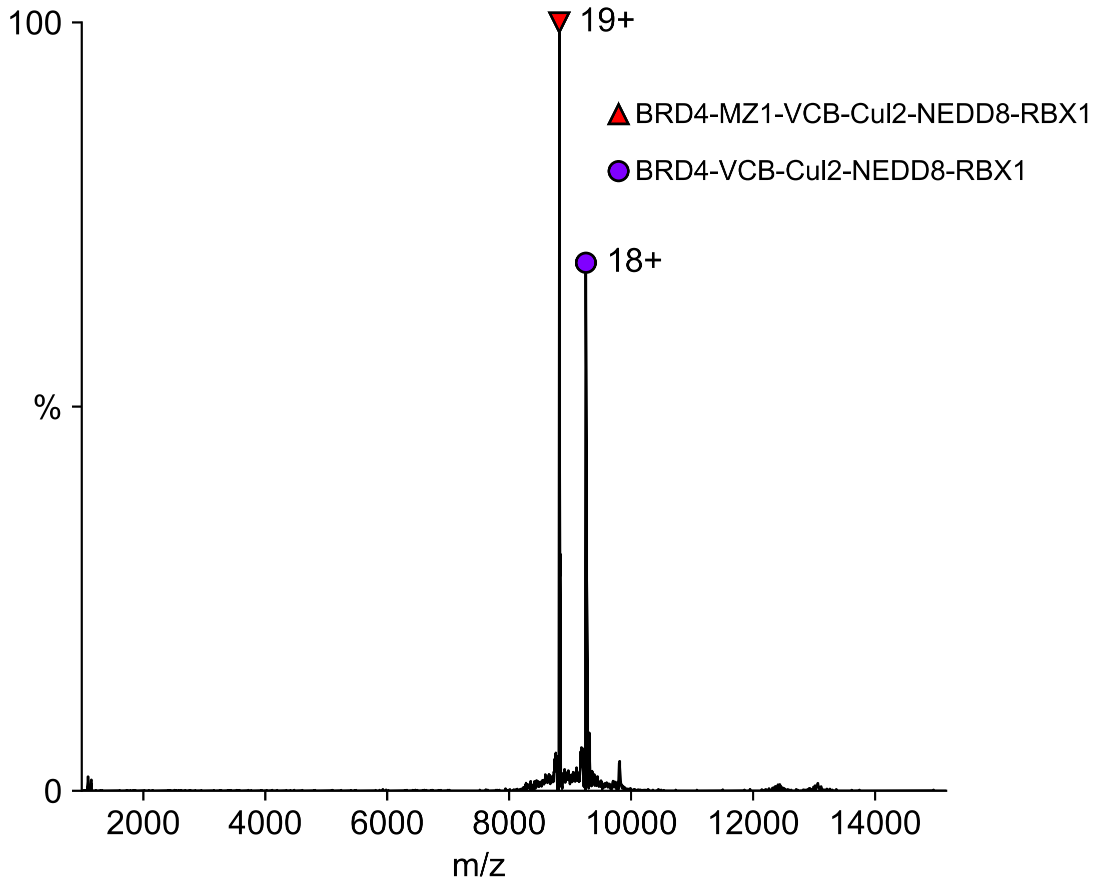

Figure S8. CID spectrum of 19+ BRD4^{BD2}-MZ1-VCB-Cul2-NEDD8-RBX1. A charge state has been assigned for each species present.

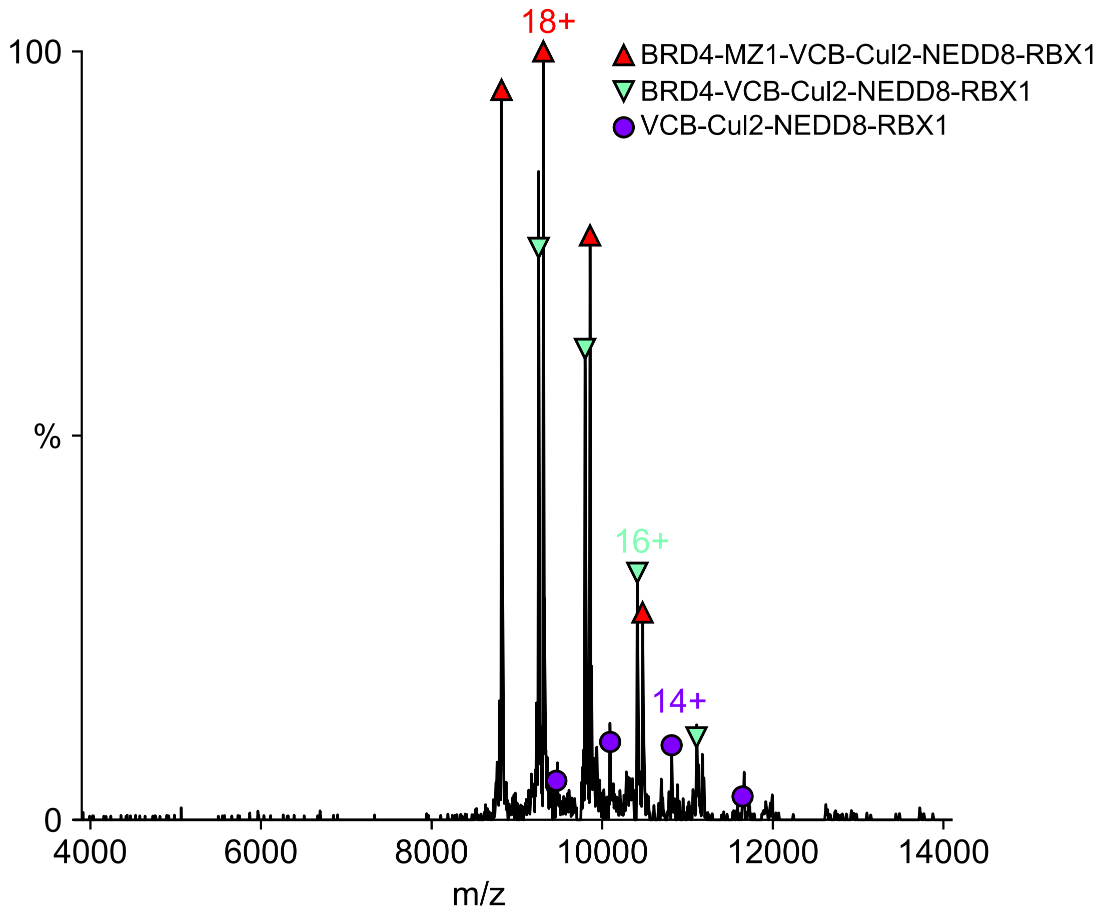

Figure S9. SID spectrum of 19+ BRD4^{BD2}-MZ1-VCB-Cul2-NEDD8-RBX1. A charge state has been assigned for each species present.

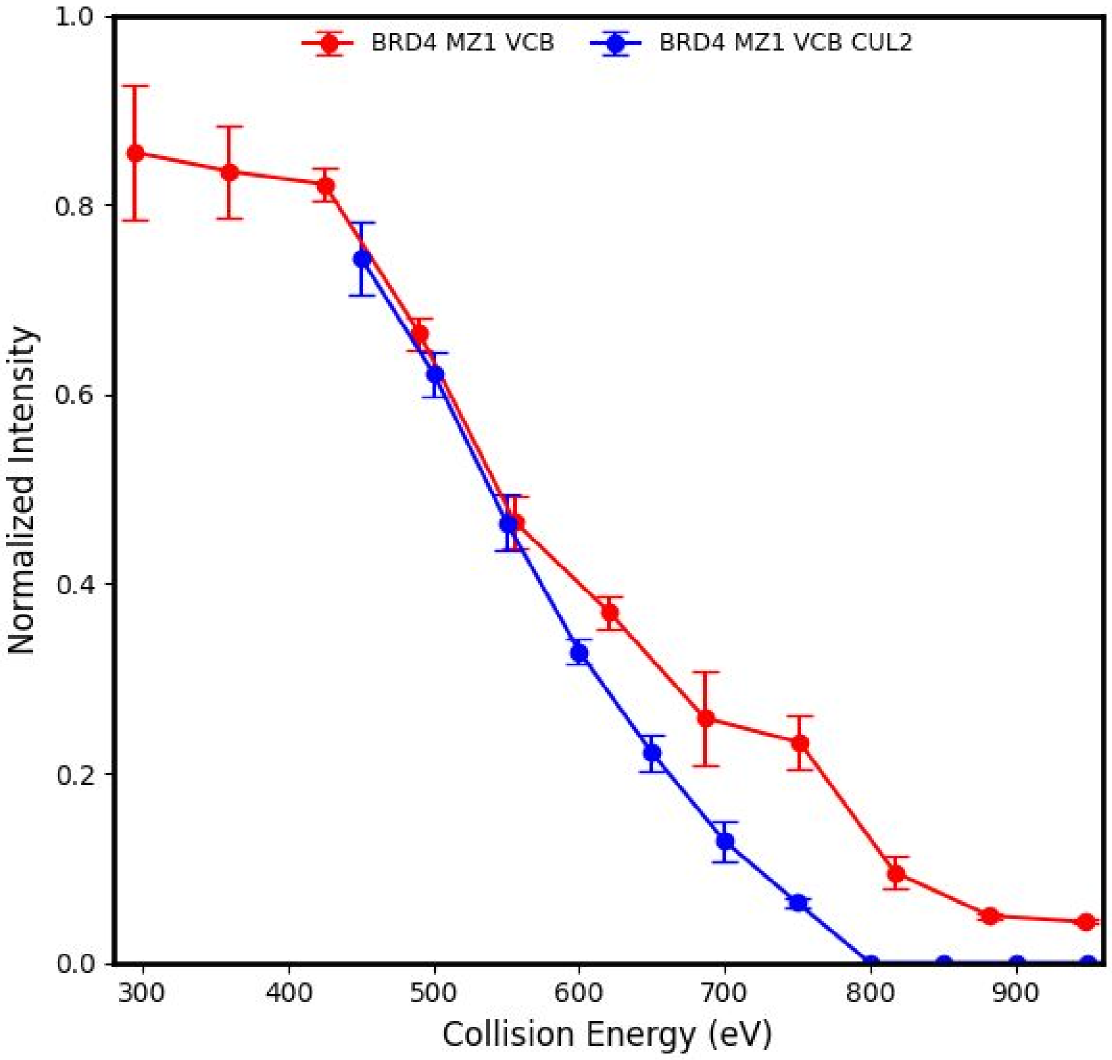

Figure S10. Precursor decay plot for 10+ BRD4^{BD2}-MZ1-VCB and 19+ BRD4^{BD2}-MZ1-VCB-Cul2-NEDD8-RBX1. The red line indicates 10+ BRD4^{BD2}-MZ1-VCB, and the blue line indicates 19+ BRD4^{BD2}-MZ1-VCB-Cul2-NEDD8-RBX1.

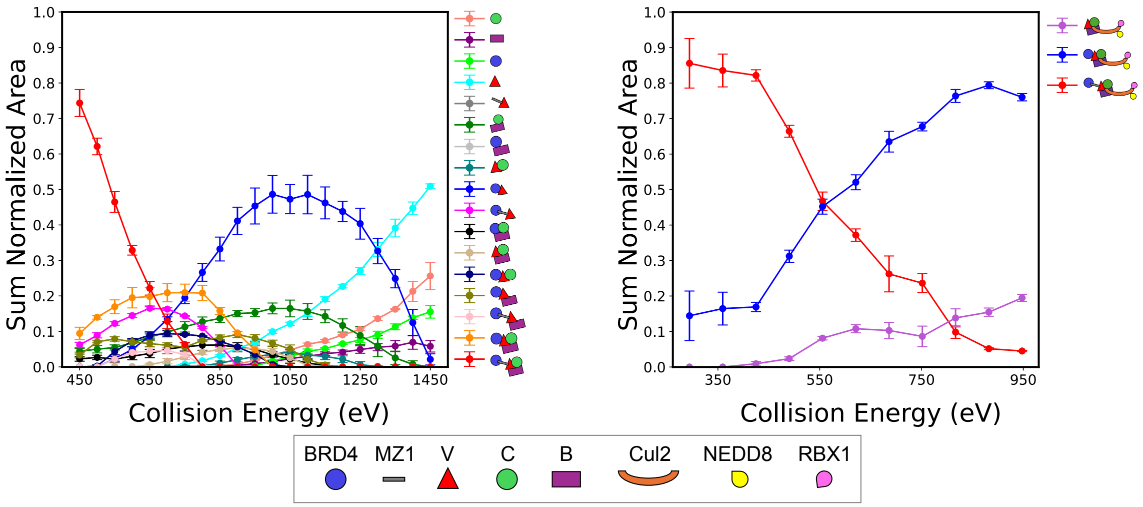

Figure S11. ERMS plots for 10+ BRD4-MZ1-VCB (left). ERMS plot for 19+ BRD4^{BD2}-MZ1-VCB-Cul2-NEDD8-RBX1 (right). The collision energies are corrected for the mass addition of Cul2.

Experimental Section :

Protein expression and purification

Gene synthesis was performed by Genscript (Piscataway, NJ). Human VHL (54-213), Elongin B (1-104), and Elongin C (17-112) proteins were co-expressed as a complex in E. coli using co-transformation of pET28 (VHL) and pCDFDuet-1 (ELOB/ELOC) vectors. VHL contained an N-terminal 6xHis tag for purification followed by a thrombin protease recognition sequence. BL21 (DE3) cells were grown in TB medium supplemented with kanamycin (100 µg/mL) and streptomycin (100 µg/mL) and induced overnight with 1mM IPTG at 18 °C. The cells were lysed with high pressure by passage through an EmulsiFlex-C5 (Avestin) and the insoluble fraction removed by centrifugation at 50000 rcf. Purification was performed by a combination of polyhistidine affinity capture using Ni Sepharose 6 FF (Cytiva), incubation with thrombin protease to cleave the tag, anion exchange chromatography with Q Sepharose HP (Cytiva), and size-exclusion chromatography using a Superdex 75 26/600 column (Cytiva) into a final buffer of 10 mM Bis-tris propane (pH 7.0), 150 mM NaCl, 5 mM DTT. Final samples were concentrated using an Amicon Ultra-15 centrifugal filter (Millipore) to 30 µM for further use.

BRD4 bromodomain 2 (352-457) was cloned into a pET28 vector with an N-terminal 8xHis tag followed by an HRV 3C protease recognition sequence and a C-terminal Avitag. The protein was expressed and purified as above using His affinity chromatography, tag cleavage with HRV 3C protease, and SEC into 25 mM HEPES (pH 7.5), 250 mM NaCl, 5% glycerol, 0.5 mM TCEP. The final sample was concentrated and stored at 100 µM.

CUL2/RBX1 complex was generated by co-expression of the proteins in insect cells. The full-length forms of N-terminally StrepII-HRV 3C tagged CUL2 (2-745) and untagged RBX1 (1-108) were cloned separately into pFastBac-1 vectors. Baculoviruses were generated using the Bac-to-Bac system (Gibco) and used to co-infect Sf9 cells grown in SF900-II medium (ThermoFisher) supplemented with 25 µM zinc acetate. The cells were then lysed by passage through a Douce homogenizer and the insoluble fraction cleared by centrifugation at 35000 rcf. The complex was then purified by batch chromatography with Strep-Tactin XT 4Flow (IBA) and SEC over a Superdex 200 26/600 column (Cytiva) equilibrated in 25 mM HEPES pH 7.5, 150 mM NaCl, 1 mM TCEP.

The resulting CUL2 protein was neddylated by in vitro enzymatic conjugation with internally purified NEDD8, NEDD8 E1 (APPBP1/UBA3 complex), and NEDD8 E2 (UBE2M), as previously described.[1] Briefly, CUL2/RBX1 at 4 µM was mixed with 35 µM NEDD8, 0.5 µM E1, 1 µM E2, and 2 mM ATP in 25 mM HEPES pH 7.5, 150 mM NaCl, 1 mM TCEP, 10 mM $MgCl_2$ for 18 hours at 4 °C. Nearly complete neddylation was confirmed by gel shift in SDS-PAGE analysis. The resulting mixture was purified by StrepII affinity resin as described above to isolate neddylated CUL2/RBX1 followed by

buffer exchange into 25 mM HEPES pH 7.5, 150 mM NaCl, 1 mM TCEP using a 10DG desalting column (BioRad). The final protein was concentrated to 14 μM prior to use.

Sample Preparation

AbbVie provided the PROTACs, BRD4, Cul2, and the VCB complex. Proteins were buffer exchanged into 200 mM ammonium acetate using BioRad 6 kDa size-exclusion spin columns. PROTACs were dissolved in DMSO prior to adding to protein samples for complex formation and diluted to a 1% (v/v) 100 μM stock in water. After the buffer exchange, a mixture of 1:2:1 BRD4:PROTAC: VCB was mixed to form the BRD4-PROTAC-VCB complex and incubated at room temperature for 15 minutes (20 μM MZ1 mixed with 10 μM $BRD4^{BD2}$ and 10 μM VCB). The complex using MZ1 was analyzed both with and without Cul2 bound. To achieve binding, Cul2 was mixed with the VCB complex at a 1:1 ratio (6 μM each) and incubated for one hour on ice. Subsequently, BRD4 and MZ1 were added in the same ratios (6 μM BRD4, 12 μM MZ1) as when forming the complex without Cul2. For all SID experiments, 20% triethyl ammonium acetate by volume was added to reduce the charge of the complex and to retain more native-like structures.[2]

Mass Spectrometry

nMS and SID-ERMS

MS experiments were performed on a Q Exactive™ UHMR Hybrid Quadrupole Orbitrap™ Mass Spectrometer (Thermo Scientific, Bremen, Germany), which has been modified with a 4-cm SID device.[3] Samples were ionized using a nano-electrospray ionization (nano-ESI) source. Aliquots of 3-5 μL of the sample were injected into borosilicate capillaries that were prepared in-house by pulling with a P-97 micropipette capillary puller (Sutter Instruments, Novato, CA). Analyses were done in positive mode with the following parameters: electrospray voltage, between 0.5 and 0.9 kV; capillary temperature, 250 °C; in-source trapping, -30 V; arbitrary trap gas flow rate at 5 (ultrahigh vacuum pressure gauge reading ~ 2.8×10^{-10} mbar); resolution was varied between 3k, 6k and 12k. ERMS plots were produced by acquiring data from tandem MS experiments with SID voltage potentials ranging from 45-145 V. Typical voltage settings and instrument parameters for SID can be found in Table S1. Mass spectra were extracted and examined with Xcalibur 4.1 (Thermo Scientific). Spectral deconvolution was carried out with UniDec software.[4] Detailed processing parameters are described in the supporting information.

References

(1) Walden, H.; Podgorski, M. S.; Huang, D. T.; Miller, D. W.; Howard, R. J.; Minor, D. L.; Holton, J. M.; Schulman, B. A. The Structure of the APPBP1-UBA3-NEDD8-ATP Complex Reveals the Basis for Selective Ubiquitin-like Protein Activation by an E1. *Molecular Cell* **2003**, *12* (6), 1427–1437. https://doi.org/10.1016/S1097-2765(03)00452-0.

(2) Zhou, M.; Dagan, S.; Wysocki, V. H. Impact of Charge State on Gas-Phase Behaviors of Noncovalent Protein Complexes in Collision Induced Dissociation and Surface Induced Dissociation. *Analyst* **2013**, *138* (5), 1353–1362. https://doi.org/10.1039/C2AN36525A.

(3) VanAernum, Z. L.; Gilbert, J. D.; Belov, M. E.; Makarov, A. A.; Horning, S. R.; Wysocki, V. H. Surface-Induced Dissociation of Noncovalent Protein Complexes in an Extended Mass Range Orbitrap Mass Spectrometer. *Anal. Chem.* **2019**, *91* (5), 3611–3618. https://doi.org/10.1021/acs.analchem.8b05605.

(4) Marty, M. T.; Baldwin, A. J.; Marklund, E. G.; Hochberg, G. K. A.; Benesch, J. L. P.; Robinson, C. V. Bayesian Deconvolution of Mass and Ion Mobility Spectra: From Binary Interactions to Polydisperse Ensembles. *Anal. Chem.* **2015**, *87* (8), 4370–4376. https://doi.org/10.1021/acs.analchem.5b00140.
